# Effect of lacosamide on cortical spreading depolarization in mice

**DOI:** 10.1186/s10194-025-02099-9

**Published:** 2025-07-16

**Authors:** Chisato Iba, Miyuki Unekawa, Keiko Ihara, Yoshikane Izawa, Jin Nakahara, Tsubasa Takizawa

**Affiliations:** Department of Neurology, Keio University School of Medicine, Tokyo, 160-8582 Japan

**Keywords:** Lacosamide, Cortical spreading depolarization, Migraine, Antiseizure medication

## Abstract

**Background:**

Antiseizure medications are often used as preventive treatment of migraine in appropriate cases; however, the efficacy of lacosamide (LCM), a sodium channel blocker, in preventing migraine attacks remains unclear. Cortical spreading depolarization (CSD) refers to a wave of slowly propagating depolarization across the cerebral cortex. CSD animal models have been extensively used to investigate migraine attacks and evaluate the effects of migraine medication. Herein, we examined the effects of single dose LCM (40 mg/kg) on CSD sensitivity in a mouse model.

**Findings:**

Thirty-two C57BL/6 mice (male, *n* = 16; female, *n* = 16) were intraperitoneally injected with either LCM (40 mg/kg) or saline before CSD sensitivity testing. Potassium chloride (KCl) was administered to induce CSD, and the CSD threshold, frequency, and propagation velocity were determined. The average CSD frequency induced by 1 M KCl was significantly lower (*p* = 0.030) and the CSD propagation velocity tended to be lower in the female LCM group than in the saline group. However, no significant differences were observed in any of the three CSD parameters in male mice.

**Conclusions:**

In female mice, single dose LCM treatment significantly reduced CSD frequency induced by KCl. Further investigations are warranted to assess the clinical potential of LCM in preventing migraine.

## Background

Migraine is one of the most prevalent neurological disorders worldwide, affecting over one billion individuals [1], and is known to be the second leading cause of years lived with disabilities (YLDs) [2, 3]. The enhancement of therapeutic interventions is crucial to improve the well-being of patients. Recent advancements in migraine treatment options such as calcitonin gene-related peptide (CGRP) monoclonal antibody therapy has started a new era in the management of this condition. However, approximately 25–30% of patients do not respond to this novel treatment [4, 5], underscoring the necessity for the development of alternative therapeutic interventions.

Conventional prophylactic medications for migraine include beta-blockers, tricyclic antidepressants, calcium channel blockers, and antiseizure medications. Among antiseizure medications, valproate and topiramate are most commonly used for migraine treatment [3, 6–8]. Lacosamide (LCM) is another antiseizure drug that enhances the slow inactivation of voltage-dependent sodium channels; it is widely used and has been approved for the treatment of epilepsy, including partial-onset seizures and tonic–clonic seizures, based on high-level evidence [9, 10]. Among antiseizure medication used for migraine treatment, valproate and topiramate are potentially teratogenic and cannot be used by pregnant women, which poses significant limitations in clinical practice since migraine is most prevalent in the young female population [11, 12]. Although only limited evidence currently exists on LCM safety during pregnancy [13, 14], this could potentially broaden treatment options in the future. Although LCM has not been approved for the treatment of migraine, there have been some reports of its analgesic effects in animals and humans. In vivo models have demonstrated that LCM dose-dependently attenuated mechanical hyperalgesia following carrageenan injection and in rats suffering from Freund's complete adjuvant-induced arthritis. Moreover, thermal hyperalgesia induced by carrageenan as well as the formalin-induced licking response were dose-dependently attenuated by LCM [15]. In addition, LCM has been reported to inhibit cephalic and extracephalic cutaneous allodynia induced by the activation of dural nociceptors with inflammatory mediators in rats [16]. LCM also has been shown to suppress CGRP release from brainstem explants both under baseline conditions and following pharmacological stimulation, as well as to reduce the CGRP levels in the brainstem and trigeminal ganglia following nitroglycerin administration [17]. In clinical settings, LCM relieved idiopathic and secondary facial pain refractory to other drugs [18]. In addition, administration of 50 mg LCM twice a day was associated with a reduction in frequency and duration of migraine in episodic patients with migraine [19].

Cortical spreading depolarization (CSD) is a wave of slowly propagating depolarization [20, 21]. CSD plays an important role in the pathophysiology of migraine, especially migraine aura. In human visual cortex, the propagation of CSD was observed using functional MRI in a pattern consistent with migraine aura [22, 23]. In addition, the pathophysiological changes associated with CSD such as cerebral blood flow and neural activity were similar to those observed in patients with migraine by functional imaging [24]. Moreover, CSD reportedly triggers the release of CGRP and inflammatory cytokines, which activate the trigeminal nervous system. These are both key mediators of migraine pain [21, 25–28]. The animal model of CSD is a well-established migraine model often used to investigate the effects of migraine treatment [21]. Thus, when attempting drug repurposing, observation of the effects of existing drugs on CSD model is a reasonable method. Although some previous studies have indicated the effect of LCM on pain, their effect on migraine have limited evidence in humans and their effect on CSD in rodent models also remains unclear. To bridge this gap in our understanding, we investigated the effects of single dose LCM treatment on a mouse CSD model induced by potassium chloride (KCl).

## Methods

### Experimental procedures

All experimental procedures were approved by the Keio University Institutional Animal Care and Use Committee (authorization no. A2021-006) and were performed in accordance with the university’s guidelines and the Animal Research: Reporting In Vivo Experiments (ARRIVE) reporting guidelines for the care and use of laboratory animals.

### Animals

Thirty-two C57BL/6 mice (age: 8-week; male: *n* = 16, female: *n* = 16) were used in this study (CLEA Japan Inc., Tokyo, Japan). The mice were housed under a 12 h dark–light cycle with free access to water and food. Both male mice and female mice were randomly divided into two groups; LCM group and saline group. On the day of the experiments, the mice were injected intraperitoneally with LCM (40 mg/kg in 5 mL/kg saline, Axon1444, Axon Medchem. Groningen, Netherland) or saline (5 mL/kg). Immediately after the injection, animals were anesthetized using isoflurane (2.0% during surgery and 1.5% during CSD evaluation).

### Cortical spreading depolarization evaluation

The protocol for investigating CSD sensitivity has been described previously [29–31]. The researcher conducting the CSD experiments (MU) was blinded to the drugs administered. A catheter was inserted into the femoral artery to measure the arterial blood pressure and heart rate. The mice were fixed to a head holder (SGM-4; Narishige Scientific Instrument Laboratory, Tokyo, Japan). Direct current (DC) electrodes (EEG-5002Ag; tip diameter = 200 µm; Bio Research Center, Aichi, Japan) were affixed to the cerebral surface of the left parietal lobe, and two locations on the right parietal and frontal lobe. Regional cerebral blood flow (rCBF) was constantly monitored in both temporoparietal regions using a laser Doppler flowmeter (ALF21; Advance Co. Ltd., Tokyo, Japan). Arterial blood gas was measured through the femoral arterial catheter before the CSD sensitivity test using Rapid Lab 348EX (Siemens AG., Munich, Germany).

**Fig. 1:**
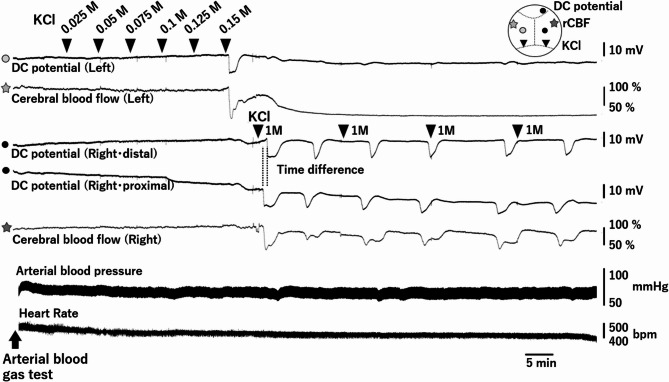
Cortical spreading depolarization (CSD) sensitivity test. Experimental protocol and setup for the CSD sensitivity test are shown. Mice were intraperitoneally injected with lacosamide (LCM) or saline prior to surgery. The figure on the top right shows the location of each probe on the skull. Laser Doppler probes (★), and DC electrodes (●) were fixed onto the surface. Two skull windows (▼) were made for KCl application. DC, direct current; rCBF, regional cerebral blood flow

After confirming the absence of CSD for at least 30 min, CSD sensitivity test was conducted. 5 µL of 0.025 M KCl solution was introduced into the left posterior window. The KCl concentration was increased by 0.025 M at intervals of at least 5 min, and the concentration at which CSD occurred for the first time was considered as the CSD “threshold”. CSD frequency was defined as the number of CSDs occurring within 60 min following the placement of cotton balls soaked in 1 M KCl on the right posterior window. The cotton balls were replaced at 15-min intervals. The propagation velocity was measured using the distance between the DC electrodes, and the detected time difference (Fig. 1).

### Statistical analysis

**Fig. 2:**
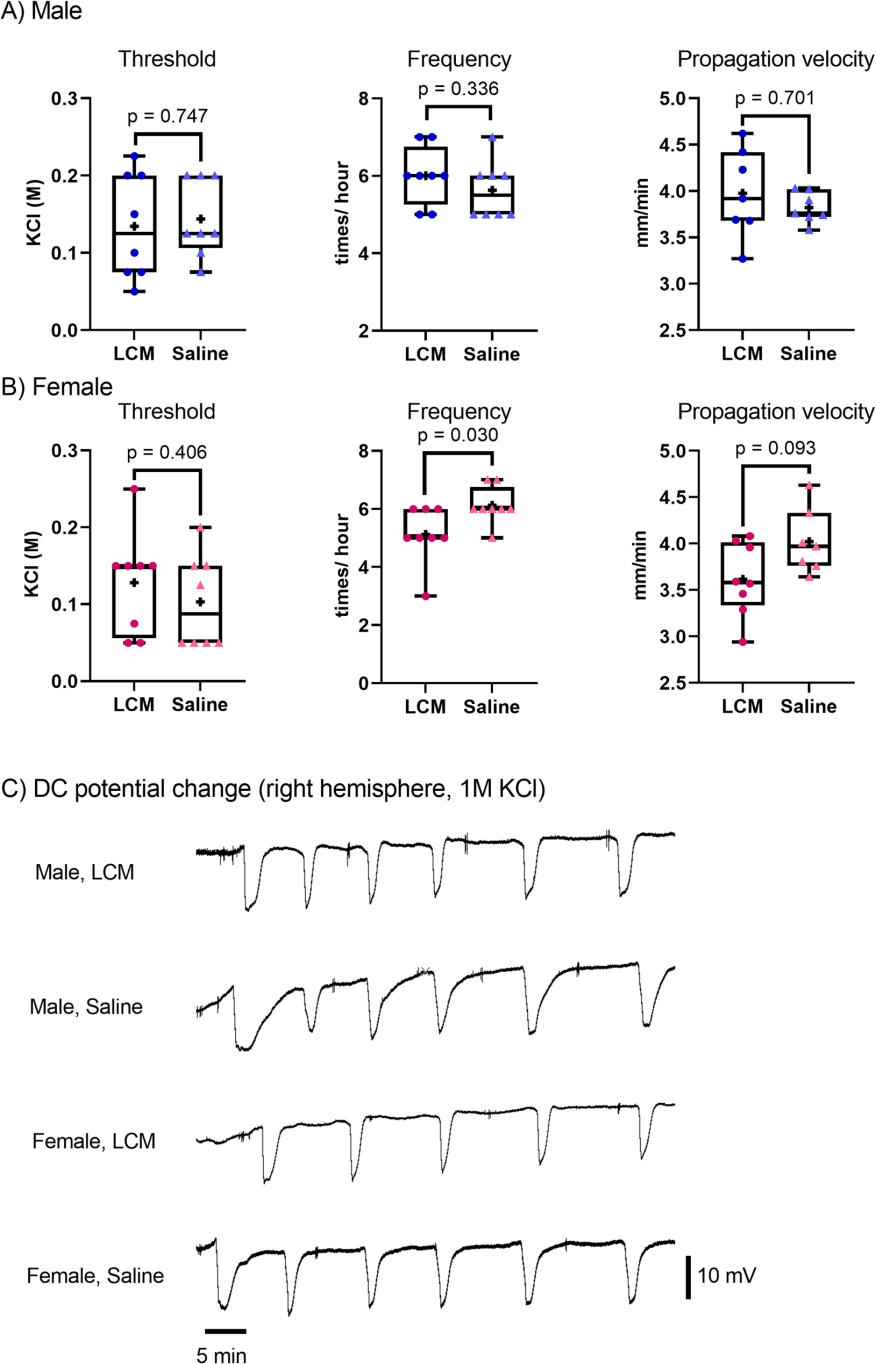
Comparison of the cortical spreading depolarization (CSD) sensitivity in male (**A**) and female (**B**) mice, and representative images of direct current (DC) potential changes in the right hemisphere at 1 M KCl (**C**). **A**, **B** Comparison of CSD sensitivity, including the CSD threshold, frequency, and propagation velocity, between mice treated with LCM and those treated with saline. Whiskers, boxes, horizontal lines, and + represent the complete range, interquartile range, median, and mean, respectively. **C** Representative images showing changes in the DC potentials following 1 M KCl application in the right hemisphere during CSD frequency measurement are presented for each group

Results are expressed as mean ± standard deviation (SD). To evaluate the CSD sensitivity, the CSD threshold, frequency at 1 M KCl, and propagation velocity were recorded. All analyses were performed using R version 4.3.2. The Shapiro -Wilk test indicated that the data were not normally distributed. The Wilcoxon rank-sum test was used to compare the CSD sensitivity and arterial blood gas analyses between the two groups. Statistical significance was set at *p* < 0.05. Figure 2 was created using GraphPad Prism version 9.5.1 for Windows (GraphPad Software, Boston, Massachusetts, USA, www.graphpad.com).

## Results

All 32 mice used in this study were included in the analyses. The mean time between LCM or saline injection and the commencement of the CSD sensitivity test was 79.3 ± 7.20 min. In male mice, arterial blood gas tests performed before the CSD sensitivity test revealed higher pH and lower MABP in the LCM group compared to the saline group. On the other hand, in female mice, the LCM group showed lower HR than the saline group (Table 1). In male mice, no significant differences in CSD parameters including threshold, frequency, and velocity, were observed. However, in female mice, CSD frequency induced by 1 M KCl was significantly lower in the LCM group than in the saline group (5.1 ± 1.0 vs. 6.1 ± 0.6, *p* = 0.030). Furthermore, propagation velocity tended to be slower in the LCM group than in the saline group, though the difference was not significant (3.62 ± 0.4 vs. 4.02 ± 0.3 mm/min, *p* = 0.093). No significant differences were observed in the CSD threshold between the two groups (0.128 ± 0.07 M vs. 0.103 ± 0.06 M, *p* = 0.406). Overall, CSD susceptibility was somewhat lower in female mice administered LCM compared to those administered saline (Figure 2). CSD parameters did not show any significant differences between male saline group and female saline group.

**Table 1:** The results of the arterial blood gas analyses performed before the cortical spreading depolarization (CSD) test in male mice (A) and female mice (B)

(A)			
Male	Lacosamide	Saline	
Body weight (g)	23.8 ± 1.5	23.1 ± 1.6	
pH	7.35 ± 0.04	7.31 ± 0.04	*
PaCO₂ (mmHg)	44.2 ± 4.4	45.1 ± 7.3	
PaO₂ (mmHg)	103.3 ± 7.0	93.8 ± 11.4	
Na (mmol/L)	146.6 ± 1.2	147.5 ± 1.2	
K (mmol/L)	4.33 ± 0.29	4.46 ± 0.23	
Hct (%)	46.3 ± 1.0	47.1 ± 1.6	
HCO₃ (mmol/L)	24.0 ± 2.1	22.2 ± 2.9	
MABP (mmHg)	76.6 ± 3.5	80.6 ± 3.2	*
HR (bpm)	461 ± 48	455 ± 34	
(B)			
Female	Lacosamide	Saline	
Body weight (g)	19.0 ± 1.1	18.9 ± 0.9	
pH	7.32 ± 0.03	7.30 ± 0.04	
PaCO₂ (mmHg)	47.0 ± 4.4	43.9 ± 5.8	
PaO₂ (mmHg)	106.6 ± 15.6	116.4 ± 13.8	
Na (mmol/L)	148.0 ± 1.3	146.3 ± 2.3	
K (mmol/L)	3.84 ± 0.50	3.80 ± 0.41	
Hct (%)	47.4 ± 1.4	47.1 ± 1.1	
HCO₃ (mmol/L)	23.4 ± 1.4	20.9 ± 1.4	
MABP (mmHg)	75.5 ± 8.8	81.8 ± 4.8	
HR (bpm)	405 ± 41	483 ± 26	*

The arterial blood gas tests showed significant changes in some parameters; higher pH and lower MABP in the lacosamide (LCM) group compared to the saline group in male mice, and the LCM group showed lower HR than the saline group in female mice. Each value represents the mean ± SD

^*^ represents the significant differences between the LCM group and the saline group

*Hct* Hematocrit, *MABP* Mean arterial blood pressure, *HR* Heart rate

## Discussion

In this study, we investigated whether a single dose of LCM affects CSD sensitivity in mice. In female mice injected with LCM, the frequency of CSD was significantly lower, and the propagation velocity tended to be slower than in the saline group, whereas LCM had no significant effect on CSD in male mice.

The effect of LCM in patients with migraine has been investigated in a clinical trial [19]. Although LCM has not been approved for the treatment of migraine, it has been used as an antiseizure medication. An open-label randomized control study investigated the serum CGRP-like immunoreactivity level (CGRP-L1) between two treatment groups: LCM and ibuprofen versus ibuprofen alone. LCM 50 mg twice a day was used as prophylaxis and ibuprofen was used as acute medication. The chronic treatment of LCM plus ibuprofen significantly reduced the CGRP-L1, which reflects the peptide whose concentration reportedly increased during migraine attacks, although with some controversies [32, 33]. As the study was designed to administer ibuprofen to patients, the effect of combining two drugs should be considered. In addition, clinical migraine parameters, including migraine frequency and duration, were also significantly reduced. LCM also has significant effects on other neurological disorders, including refractory facial pain and diabetic neuropathy [34, 35]. Although the present study demonstrated an inhibitory effect of CSD in female mice, further investigation is necessary to elucidate the precise pharmacological mechanisms underlying the potentially therapeutic effects of LCM during migraine attacks.

The complicated pathophysiology of migraine has been investigated by several animal models of migraine, such as the hyperalgesia model using repeated nitroglycerin injection and the recurrent trigeminovascular activation model. While CSD is often regarded as a model that mimics the pathophysiology of migraine with aura, the hyperalgesia model is considered to be related to allodynia [36]. Although we only investigated the effect of LCM on the CSD model, further experiments using other migraine models, such as the hyperalgesia model, would provide a broader insight into the efficacy of LCM in migraine and its mechanism in migraine pathophysiology.

In our experiments, a single dose of 40 mg/kg LCM was administered, following the methodology outlined in previous literature [15, 37]. Studies have demonstrated that LCM exerted an inhibitory effect on mechanical hyperalgesia at doses of 10–40 mg/kg, and on secondary generalized seizures at a dose of 20 mg/kg. Although it is possible that higher doses of LCM might alter other parameters of CSD, we considered 40 mg/kg to be a sufficiently high dose for investigation based on the existing literature. Several studies have investigated migraine prevention strategies in animal models of CSD. Gabapentin demonstrated an inhibitory effect on CSD after acute treatment, whereas for valproate and amitriptyline, only chronic treatment showed consistent inhibition of CSD [21]. Studies on the effects of antiseizure medications on CSD are summarized in Table 2 [38–46].

**Table 2:** Effect of antiseizure medications on CSD

Medication	Mechanism of action	Ref	CSD induction	Administration timing	CSD parameter	results
Valproate*	Inhibits voltage-dependent Na^+^ and T-type Ca^2+^ channel, enhances GABA transmission	[39]	mechanical stimulation	immediately before	rate of propagation, CBF increase	a: -
		[40]	KCl	60 min before or daily for 3 to 16 weeks	frequency, threshold, speed	a: - c: +
		[41]	KCl or electrical stimulation	60 min before or daily for 5 weeks	frequency, threshold, speed, propagation failure	a: - c: +
		[42]	KCl	daily for 4 weeks	frequency, speed, and number of Fos-immunoreactive nuclei	c: +
Topiramate*	Inhibits Na^+^ channels, kainite receptors and carbonic anhydrase. Enhances GABA_A_	[40]	KCl	60 min before or daily for 1 to 17 weeks	frequency, threshold, speed	a: - c: +
		[43]	mechanical stimulation	30 min before	rCBF, electrical nerve cell activity	a: +
		[44]	KCl	daily for 6 weeks	frequency, intervals, total time, speed	c: +
		[45]	KCl	drug incubation (ex* vivo)*	area covered by the propagation	a: -
Carbamazepine	Enhances Na^+^ channel rapid inactivation, block L-type Ca^2+^ channel	[45]	KCl	drug incubation (ex *vivo*)	area covered by the propagation	a: -
Oxcarbazepine	Enhances Na^+^ channels rapid inactivation; blocks HCav_3.2_ Ca^2+^ channel; enhances K^+^ conductance	[41]	KCl or electrical stimulation	90 min before or twice a day for 5 weeks	frequency, threshold, speed, propagation failure	a: - c: -
Gabapentin	Binds pre-synaptic α_2_-δ subunit of Ca^2+^ channel to modulate Ca^2+^ current	[46]	KCl or electrical stimulation	60 min before	threshold, frequency	a: +
Lamotrigine	Enhances Na^+^ channel rapid inactivation: Inhibits Ca^2+^ channels	[42]	KCl	daily for 4 weeks	frequency, speed, and number of Fos-immunoreactive nuclei	c: +
Lacosamide	Enhances Na^+^ channel slow inactivation		KCl	about 80 min before	frequency, threshold, speed	a: +

Antiseizure medications whose effect on animal CSD models have been investigated are summarized along with current study. Mechanism of action of each medication are cited from"Current Review in clinical science -Summary of Antiepileptic Drugs Available in the United States of America” [38]

*: antiseizure medications used for migraine prevention. Ref: reference; a: results of acute experiments; c: results of chronic experiments; +: the treatment showed suppressive effect on CSD in at least one of the parameters; - : the treatment showed no suppressive effect on CSD

Although we determined that single dose LCM resulted in a significant change in the CSD frequency in female mice [47], we cannot rule out the possibility that long-term treatment of LCM may lead to changes in other parameters in females or even in males. Given that migraine preventive medications are prescribed for at least a few months in clinical practice, it remains imperative to investigate consecutive treatment of LCM in future studies.

Sex differences could also influence CSD sensitivity. In our results, all the CSD parameters did not show any significant difference between the male saline group and female saline group. However, at the baseline, one of the previous studies demonstrated reduced CSD threshold in female mice [48], which is hypothesized to be attributed to the differences in the sex hormones [49, 50]. Although clinical trials of LCM have not explicitly addressed the issue of sex-related differences in drug reaction [19], there may be some variations in CSD sensitivity to drugs [51]. Additionally, female mice were suggested to have a higher susceptibility to CSD than male mice [48]. A previous study using a passive smoking model showed no changes in males and a significantly reduced CSD threshold in females, which is consistent with the present study where changes were observed only in females [30].

LCM enhances the slow inactivation of voltage-dependent sodium channels and also binds collapsin response mediator protein-2 (CRMP-2) [52]. This results in the stabilization of hyperexcitable neuronal membranes and neuronal firing, thereby inducing anti-epileptic effects. CSD might be inhibited when voltage-dependent sodium channels are deactivated by LCM. Previous studies have investigated the effects of antiseizure medications similar to LCM, which targets sodium channels. Among these, acute treatment with carbamazepine [45] or acute and chronic treatment with oxcarbazepine [41] did not alter CSD susceptibility, whereas chronic treatment with lamotrigine [42] suppressed CSD in rat models. Lamotrigine reportedly reduces migraine aura in patients with migraine, suggesting that the medication has an inhibitory effect on CSD [53, 54]. Differences may exist in their effects on animal CSD models among sodium channel-targeting antiseizure drugs. Further investigation of each sodium channel-targeting antiseizure medications would be fundamental to clarify the mechanism and clinical effects.

This study had several limitations. First, it only investigated the effects of single dose LCM on CSD, although prophylactic medicine including LCM is usually used consecutively for migraine in clinical practice. Furthermore, this study addresses only a portion of migraine pathology which is related to CSD.

In conclusion, single dose treatment of LCM had an inhibitory effect on CSD frequency in female mice, which is consistent with previous clinical studies. This demonstrates its clinical potential as migraine prophylaxis, and further studies are required to elucidate the underlying mechanisms.

## Data Availability

No datasets were generated or analysed during the current study.

## Abbreviations

LCM: Lacosamide
CSD: Cortical spreading depolarization
KCl: Potassium chloride
MABP: Mean arterial blood pressure
DC: Direct current
rCBF: Regional cerebral blood flow
HR: Heart rate
